# Isolation and genotyping of *Acanthamoeba* spp. from *Acanthamoeba* meningitis/ meningoencephalitis (AME) patients in India

**DOI:** 10.1186/s13071-016-1729-5

**Published:** 2016-08-09

**Authors:** Himanshu Sekhar Behera, Gita Satpathy, Manjari Tripathi

**Affiliations:** 1 Ocular Microbiology, Dr. R. P. Centre for Ophthalmic Sciences, All India Institute of Medical Sciences, New Delhi, 110029 India; 2 Neurosciences Centre, All India Institute of Medical Sciences, New Delhi, 110029 India

**Keywords:** *Acanthamoeba* meningoencephalitis, *Acanthamoeba* meningitis, Genotyping, Culture of *Acanthamoeba*

## Abstract

**Background:**

*Acanthamoeba* spp. are free-living ubiquitous protozoans capable of causing *Acanthamoeba* meningitis/meningoencephalitis (AME) of the central nervous system in humans. *Acanthamoeba* spp. are divided into 20 different genotypes (T1–T20) on the basis of variation in nucleotide sequences of the 18S rRNA gene. The objective of this study was to identify the genotypes of *Acanthamoeba* spp. in patients of *Acanthamoeba* meningitis/meningoencephalitis (AME) using 18S rRNA gene-based PCR assay. The present study provides information regarding the involvement of the most prevalent and predominant genotype of *Acanthamoeba* spp. in *Acanthamoeba* meningitis/meningoencephalitis infections in India.

**Methods:**

Cerebrospinal fluid (CSF) was collected from 149 clinically suspected *Acanthamoeba* meningitis/meningoencephalitis (AME) patients reporting to the outpatient department/causality services of the Neurosciences Centre, AIIMS, New Delhi, India during the past five years. Samples were inoculated onto 2 % non-nutrient agar plates overlaid with *E. coli* and incubated at 30 °C for 14 days. Among 149 suspected patients, ten were found culture-positive for *Acanthamoeba* spp. out of which six isolates were established in axenic culture for molecular analysis. DNA was isolated and a PCR assay was performed for amplification of the Diagnostic fragment 3 (DF3) (~280 bp) region of the 18S rRNA gene from axenic culture of six *Acanthamoeba* spp. isolates*. Rns* genotyping was performed on the basis of the variation in nucleotide sequences of DF3 region of the 18S rRNA gene.

**Results:**

In the phylogenetic analysis, all of the six *Acanthamoeba* spp. isolates were found to belong to genotype T4. The sequence homology search for these six isolates in the NCBI databank showed homology with the available strains of *Acanthamoeba* spp*.* The newly generated sequences are available in the GenBank database under accession numbers KT004416–KT004421.

**Conclusions:**

In the present study, genotype T4 was found as the most prevalent and predominant genotype in *Acanthamoeba* meningitis/ meningoencephalitis infections. Hence further studies are needed to develop optimal therapeutic strategy against *Acanthamoeba* spp. of genotype T4 to combat against the infections.

## Background

*Acanthamoeba* spp. are free-living ubiquitous protozoans capable of causing *Acanthamoeba* meningitis/meningoencephalitis (AME) of the central nervous system and fatal granulomatous amoebic encephalitis (GAE) of the brain in humans [1]. AME is a slow progressive infection of the central nervous system that occurs mostly in immunocompromised individuals with HIV/AIDS, tuberculosis, systemic lupus erythematosis (SLE), diabetes or undergoing cancer chemotherapy/radiotherapy treatment and can also occur in immunocompetent individuals [2–5]. Although a person may be infected with *Acanthamoeba* spp., the severity of infection depends on the size of the inoculum, the immunity of the patient and the virulence properties of the infective amoeba strain. AME results from the haematogenous spread of the amoebae from initial portals of entry, i.e. either skin or respiratory system to brain parenchyma through olfactory nerve [6]. The clinical symptoms of AME resemble viral, bacterial or tubercular meningitis such as fever, headache, stiff neck, lethargy, vomiting, nausea, etc. [7]. With advancement of the disease, seizures, behavioural changes like diplopia, aphasia, ataxia, altered mental state and lethargy were seen as other major symptoms [7].

The first case of amoebic meningitis in a 9 year-old boy was reported by Fowler & Carter in 1965 [8]. The first case of amoebic meningoencephalitis in two Indian children was reported by Pan et al. in 1971 [9]. Thereafter, several cases of AME had been reported from various parts of India [10, 11].

Previously *Acanthamoeba* spp. was divided into three groups based on morphological characteristics i.e. cyst size, shape and growth temperature conditions which were confusing [12]. However with the advancement of molecular biology techniques, researchers could differentiate *Acanthamoeba* spp. into 20 different genotypes (T1–T20) based on the variation in nucleotide sequences of the 18S rRNA gene [13]. These genotypes are discriminated from one another by a minimum of 5 % sequence divergence and 0–4.3 % sequence dissimilarity exists within genotype T4 [14, 15]. Although genotypes T1, T2, T4, T5, T10 and T12 are responsible for causing meningitis in humans, yet genotype T4 predominates among them [16–18]. PCR assay for amplification of a 464 bp region termed as “*Acanthamoeba* specific amplimer (ASA.S1)”, within the 18S rRNA gene was found suitable for genotyping of *Acanthamoeba* spp. [19]. ASA.S1 region includes two regions, “stem 29” is the conserved region and “stem 29-1”, a ~280 bp long highly variable region is designated as Diagnostic fragment 3 (DF3) [20]. *Rns* genotyping of *Acanthamoeba* spp. with variation in nucleotide sequences of small DF3 region was found to be as robust as based on long ASA.S1 region (19). The present study aimed to determine the prevailing genotypes of *Acanthamoeba* spp. in amoebic meningitis/meningoencephalitis infections in humans.

## Methods

### Collection of clinical specimens

After informed consent and thorough clinical examination, cerebrospinal fluid was collected by the neurologists from 149 clinically suspected *Acanthamoeba* meningitis/meningoencephalitis patients with the following symptoms: headache, stiff neck, lethargy, vomiting, nausea, reporting to the Outpatient Department/ causality services of Neurosciences Centre, All India Institute of Medical Sciences, New Delhi, India in the past five years. Some patients were also having symptoms such as seizures or aphasia or ataxia or altered mental state and lethargy that were similar to symptoms in *Acanthamoeba* infections. A part of the specimen was inoculated onto a 2 % non-nutrient agar plate overlaid with *E. coli* for culturing and a second part of the specimen was placed in 500 μl PBS buffer (pH 7.4) for molecular diagnosis by PCR assay. Inoculated non-nutrient agar plates were sealed incubated at 30 °C for 14 days. Plates were examined regularly for 14 days post-incubation under a light microscope (at a magnification of 100–400×) (Nikon) for the appearance of growth of *Acanthamoeba* spp. over the agar surface. Among 149 suspected patients of *Acanthamoeba* meningitis/meningoencephalitis 10 (6.71 %) cases were found culture positive, 11 (7.38 %) cases were found PCR positive and 10 (6.71 %) cases were positive by both culture and PCR assay for *Acanthamoeba* spp. Six samples out of ten culture-positive cases were further sub-cultured for molecular analysis by transferring a square shape agar surface with *Acanthamoeba* spp. on to new agar plates previously seeded with *E. coli.*

### Establishment of axenic culture for *Acanthamoeba* spp. isolates

Axenic culture was established in 6 *Acanthamoeba* spp. isolates among 10 culture positive isolates following the procedure as described earlier [21]. We were unable to establish axenic culture in other four positive isolates due to fungal contamination. Briefly, Petri plates containing 2 % non-nutrient agar medium overlaid with live *E. coli* were treated under UV light in a laminar hood for 30 mins to kill the live bacteria. A small piece of culture of *Acanthamoeba* spp. from the previously cultured plate was placed face down on the surface of the UV treated plate and incubated at 30 °C for 7 days. Subsequently a small piece of agar containing amoebas from the UV-treated plate was transferred to 25 cm^2^ cell culture flasks (Nunc) containing 10 ml of PYG growth medium [proteosep peptone (0.75 %), yeast extract (0.75 %) and glucose (1.5 %)] (pH = 7.4) with antibiotics (penicillin and streptomycin) in bactericidal concentrations and incubated at 30 °C for 5 days [21]. Flasks were examined daily for 5–10 days under an inverted microscope (Nikon) until full growth of amoebas was seen in the medium. Initial culture flasks were subsequently sub cultured in 25 cm^2^ cell culture flasks containing PYG medium with antibiotics for at least 3 consecutive passages to achieve complete axenization.

### PCR assay

Briefly, axenic culture of 6 *Acanthamoeba* spp. isolates were centrifuged at 500× *g* for 10 min followed by washing the pellets with PBS buffer (pH 7.4) to make it free from remaining culture medium. DNA was extracted from the pellet using QIAmp DNA Mini Kit (QIAgen) following the manufacturer’s instructions and used for PCR assay. PCR amplification of the Diagnostic fragment 3 (DF3) (~280 bp) of the 18S rRNA gene of *Acanthamoeba* spp. was performed using the genus-specific forward primer 892C (5'-GTC AGA GGT GAA ATT CTT GG-3') and reverse primer JDP2 (5'-TCT CAC AAG CTG CTA GGG GAG TCA-3') [20, 22]. PCR amplification was carried out in 25 μl of final reaction volume containing 1× reaction buffer (Fermentas), 0.2 mM dNTPs (Fermentas), 0.40 μM of each primer and 1.25U Taq polymerase (Fermentas). The temperature profile of the PCR assay was as follows: initial denaturation for 10 min at 94 °C, followed by 35 cycles of denaturation for 1 min at 94 °C, primer annealing for 1 min at 57 °C, strand elongation for 1 min at 72 °C, with the final elongation for 10mins at 72 °C. DNA isolated from known isolates of *Acanthamoeba* spp. was used as a positive control and reaction mixture with 5 μl of distilled water was used as a negative control in the PCR reaction. Amplified PCR products were electrophoresed on 1.5 % agarose gel, which was visualised under a Gel documentation system (Syngene).

### Sequence homology analysis and construction of phylogenetic tree

Amplified DNA bands for DF3 region (~280 bp in length) were cut from the agarose gel and DNA was extracted using QIAquick Gel Extraction Kit (QIAgen) as per the manufacturer’s instructions. Nucleotide sequences of the purified DNA were determined commercially (Biolink) using the PCR primers 892C and JDP2 (sequences described above). Nucleotide sequences of the DF3 region of 6 *Acanthamoeba* spp. from this study were searched for homology analysis with available sequences found in the GenBank database with NCBI BLAST computer programme (NCBI, USA) and DF3 nucleotide sequences of all existing genotypes of *Acanthamoeba* spp. were retrieved from the NCBI databank (http://www.ncbi.nlm.nih.gov/pubmed). Nucleotide sequences from the present study and the homologous sequences from reference strains were analysed using MEGA6 computer programme [23]. Sequences were aligned (both pair wise and multiple sequence wise) using CLUSTAL W alignment programme implemented in MEGA6 [23]. The phylogenetic tree was reconstructed using Kimura two-parameter distance algorithm with 1000 bootstrap replicates. *Acanthamoeba* spp. strain V006 (T1 genotype) was used to root the tree. The tree was generated using the neighbour-joining method.

## Results

**Fig. 1:**
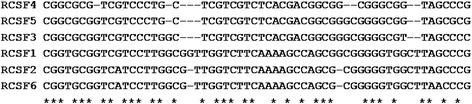
Primary sequence alignment of the DF3 region with the software CLUSTAL W. The region shown is a subset of the total DF3 region which shows highest variation. Asterisks (*) denote identical nucleotides, dashes (−) denote alignment gaps

**Fig. 2:**
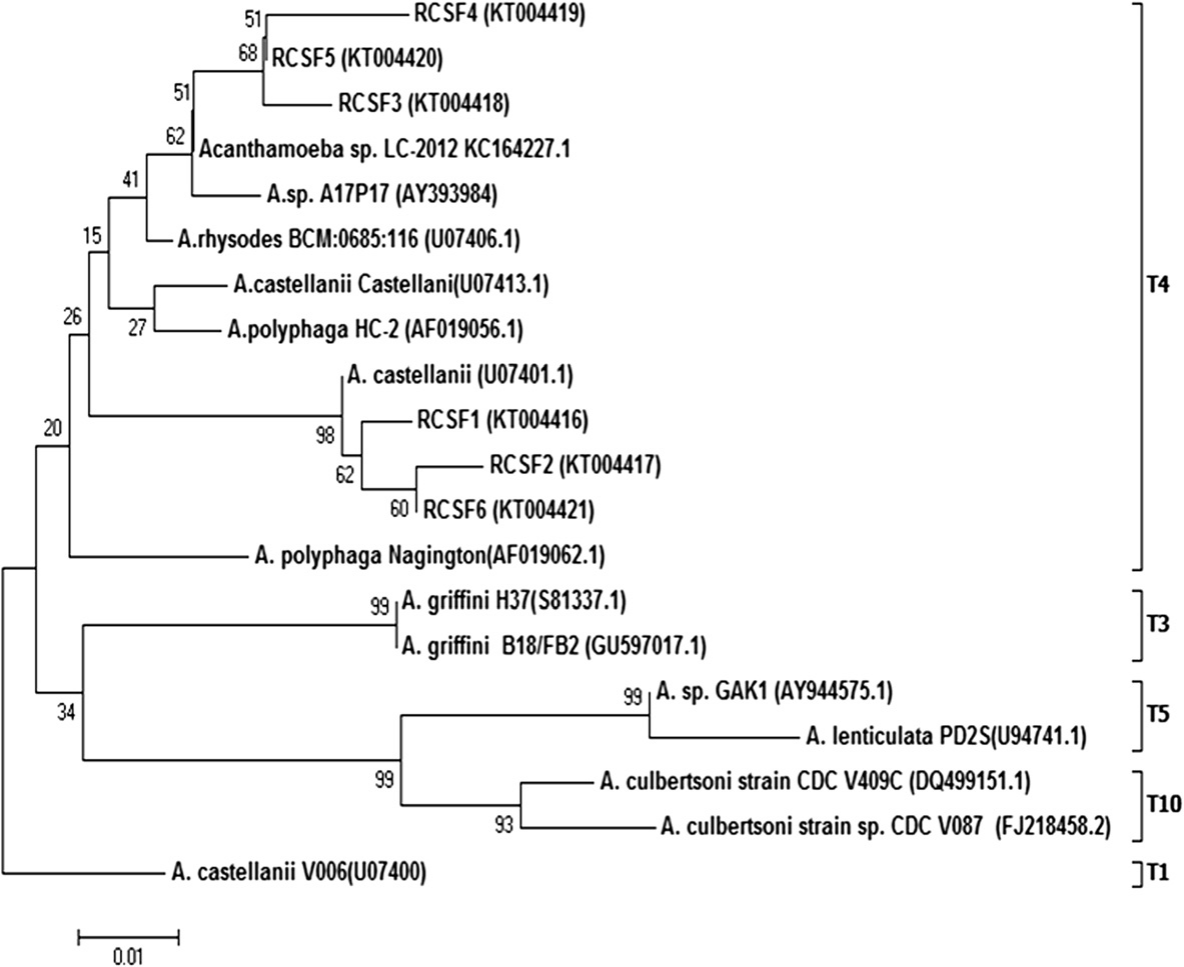
Phylogenetic tree constructed with the neighbour-joining method using nucleotide sequences of DF3 region of the 18S rRNA gene

**Fig. 3:**
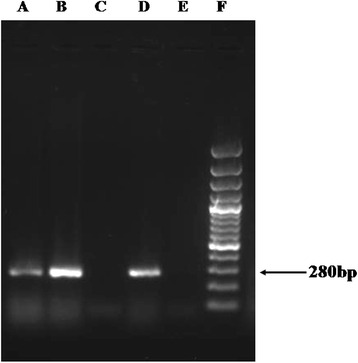
Representative photograph of 1.5 % agarose gel showing the amplified product of ~280 bp region (DF3) of the 18S rRNA gene of *Acanthamoeba* spp. Lane A: positive isolate; Lane B: positive isolate; Lane C: negative isolate; Lane D : positive control; Lane E: negative control (distiled water); Lane F: DNA ladder (100 bp)

The partial nucleotide sequences of DF3 region of *Acanthamoeba* spp. from six *Acanthamoeba* meningitis/ meningoencephalitis patients (RCSF1–RCSF6) aligned using ClustalW and showing highest variation are shown in Fig. 1. Sequence homology search for these six *Acanthamoeba* spp. in the NCBI databank revealed homology with the available strains of *Acanthamoeba* spp. presented in Table 1*.* The sequences generated in the present study are submitted to the GenBank database under accession numbers KT004416–KT004421 (Fig. 2). Nucleotide sequences for the DF3 region produced a clear band of ~280 bp length when electrophoresed in 1.5 % agarose gel for all positive specimens (Fig. 3).

**Table 1:** Comparison of *Acanthamoeba* spp. isolates obtained from six *Acanthamoeba* meningitis/ meningoencephalitis patients with reference strains available in the GenBank database

Isolates	Accession number	Genotype	Name of strain with highest homology	Accession number	Region of origin
RCSF1	KT004416	T4	AcaL7	KJ094680	Italy
RCSF2	KT004417	T4	Ac_E4c	GU808286	Thailand
RCSF3	KT004418	T4	LC-2012	KC164227	Switzerland
RCSF4	KT004419	T4	AG-2012	JQ678632	Spain
RCSF5	KT004420	T4	LC-2012	KC164227	Switzerland
RCSF6	KT004421	T4	Ac_E4c	GU808286	Thailand

Phylogenetic tree reconstructions with neighbour-joining and UPGMA methods provided similar tree topologies, which placed the six *Acanthamoeba* spp. examined here within the genotype T4 clade (Fig. 2). We found that, RCSF1 showed 100 % similarity with the previously identified strain AcaL7 of genotype T4; RCSF2 showed 100 % similarity with strain Ac_E4c of genotype T4; RCSF3 showed 97 % similarity with strain LC-2012 of genotype T4; RCSF4 showed 97 % similarity with AG-2012 of genotype T4; RCSF5 showed 99 % similarity with strain LC-2012 of genotype T4 and RCSF6 showed 99 % similarity with strain Ac_E4c of genotype T4. Out of the six *Acanthamoeba* spp. isolates, four were not 100 % identical to any available strain in the GeneBank revealing that, certain polymorphisms exists within the nucleotide sequences.

## Discussion

Free-living amoebae of the genus *Acanthamoeba* are widely distributed in nature, in the soil, water and air [7]. These are responsible for several central nervous system infections such as *Acanthamoeba* meningitis/meningoencephalitis (AME) and fatal granulomatous amoebic encephalitis (GAE). Acanthamoebae were first reported as the causative agents of acute pyogenic meningitis in 1965, and thereafter several cases of *Acanthamoeba* infections have been recorded worldwide [8, 16, 24]. Martinez & Visvesvara [6] reported that, out of 166 cases of amoebic encephalitis taken for consideration, 103 were due to *Acanthamoeba* and 63 were due to *Balamuthia*. Opportunistic infections due to *Acanthamoeba* are increasing nowadays due to decrease in immunity of the individuals mainly because of either HIV/AIDS infections or organ transplantation or chemotherapeutic treatments or administration of steroids or other debilitating diseases.

Previously *Acanthamoeba* spp. comprised three morphological groups (groups I–III) based on morphological characteristics (cyst size, shape and growth temperature requirements), which were difficult and confusing [12]. Later genotyping of *Acanthamoeba* spp. was introduced, using analysis of complete nucleotide sequences of the 18S rRNA gene, of 2,300–2,700 bp in length [19]. GTSA.B1, a large region of the 18S rRNA gene of ~1475 bp in length (approximately 65 % of the complete 18S rRNA gene), was amplified using the primers CRN5 and 1137, which was proven reliable as that of the complete 18S rRNA gene for genotyping [19]. Subsequently “ASA.S1” region, a 460 bp region within the 18S rRNA gene, was found useful as that of complete nucleotide sequences for genotyping [19]. Schroder et al. also showed that, instead of sequencing whole regions of GTSA.B1, which include six variable regions, sequencing of only one small variable region, i.e. *Acanthamoeba* Specific Amplimer (ASA.S1) of ~464 bp in length, was an useful substitute for genotyping [19]. Thereafter the DF3 region, the ~280 bp variable region within ASA.S1 region, is being widely used for genotyping studies, since it provides equivalent results as that of ASA.S1 [20].

Among all of the known 20 genotypes (T1–T20) of *Acanthamoeba* spp. T4 is the most predominant and prevalent genotype responsible for causing *Acanthamoeba* meningitis/meningoencephalitis (AME) followed by genotypes T1, T10, and T12(16). The genotype data obtained in this study from six *Acanthamoeba* spp. isolates further confirmed that, “T4 genotype is the most common and predominant genotype causing *Acanthamoeba* meningitis/ meningoencephalitis” as postulated from previous studies [16, 24]. In addition to T4, several genotypes of *Acanthamoeba* spp. were also reported from different countries from patients with meningitis or granulomatous amoebic encephalitis. In a recent phylogenetic study, *Acanthamoeba* spp. of genotypes with T2, T4 and T5 were isolated from patients with GAE [18]. Booton et al. reported that, *Acanthamoeba* spp. of genotypes T1, T10 and T12 were also responsible for causing GAE in humans [16]. *Acanthamoeba* of genotype T2 was identified as the causative agent of GAE in an immunodeficiency virus-negative patient with underlying tuberculosis [17]. Recently, *Acanthamoeba* spp. isolates of genotype T18 has been isolated from a patient with fatal GAE [25].

## Conclusions

This study supports the conclusion that, among all of the genotypes of *Acanthamoeba* spp. reported to date, T4 appears as the most prevalent and predominant genotype associated with *Acanthamoeba* meningitis/meningoencephalitis infections. Further research is needed in order to develop the optimal therapy against the T4 genotype of *Acanthamoeba* spp. to combat these fatal brain infections.

## Abbreviations

AME, *Acanthamoeba* meningoencephalitis; CSF, cerebro spinal fluid; GAE, granulomatous amoebic encephalitis

## References

[1] Marciano-Cabral F (2003). *Acanthamoeba* spp. as agents of disease in humans. Clin. Microbiol Rev.

[2] Harwood CR, Rich GE, McAleer R, Cherian G (1988). Isolation of *Acanthamoeba* from a cerebral abscess. Med J Aust.

[3] Shirwadkar CG, Samant R, Sankhe M (2006). *Acanthamoeba* encephalitis in patient with systemic lupus, India. Emerg Infect Dis.

[4] Lalitha MK, Anandi V, Srivastava A, Thomas K, Cherian AM, Chandi SM (1985). Isolation of *Acanthamoeba culbertsoni* from a patient with meningitis. J Clin Microbiol.

[5] Ringsted J, Jager BV, Suk D, Visvesvara GS (1976). Probable *Acanthamoeba* meningoencephalitis in a Korean child. Am J Clin Pathol.

[6] Martinez AJ, Visvesvara GS (1997). Free-living, amphizoic and opportunistic amebas. Brain Pathol.

[7] Visvesvara GS, Moura H, Schuster L (2007). Pathogenic and opportunistic free-living amoebae: *Acanthamoeba* spp., *Balamuthia mandrillaris*, *Naegleria fowleri*, and *Sappinia diploidea*. FEMS Immunol Med Microbiol.

[8] Fowler M, Carter RF (1965). Acute pyogenic meningitis probably due to *Acanthamoeba* sp.: a preliminary report. Br Med J.

[9] Pan NR, Ghosh TN (1971). Primary amoebic meningoencephalitis in two Indian children. J Indian Med Assoc.

[10] Sharma PP, Gupta P, Murali MV, Ramachandran VG (1993). Primary amoebic meningoencephalitis caused by *Acanthamoeba*: successfully treated with cotrimoxazole. Indian Pediatr.

[11] Singhal T, Bajpai A, Kalra V (2001). Successful treatment of *Acanthamoeba* meningitis with combination oral antimicrobials. Pediatr Infect Dis J.

[12] Pussard M, Pons R (1977). Morphologies de la paroi kystique et taxonomie du genre *Acanthamoeba* (Protozoa: Amoeba). Protistologica.

[13] Fuerst PA, Booton GC, Crary M (2015). Phylogenetic analysis and the evolution of the 18S rRNA gene typing system of *Acanthamoeba*. J Eukaryot Microbiol.

[14] Gast RJ, Ledee DR, Fuerst PA, Byers TJ (1996). Subgenus systematics of *Acanthamoeba*: four nuclear 18S rDNA sequence types. J Eukaryot Microbiol.

[15] Stothard DR, Schroeder-Diedrich JM, Awwad MH (1998). The evolutionary history of the genus *Acanthamoeba* and the identification of eight new 18S rRNA gene sequence types. J Eukaryot Microbiol.

[16] Booton GC, Visvesvara GS, Byers TJ (2005). Identification and distribution of *Acanthamoeba* species Genotypes associated with non keratitis infections. J Clinical Microbiol.

[17] Walochnik J, Aichelburg A, Assadian O (2008). Granulomatous amoebic encephalitis caused by *Acanthamoeba* amoebae of genotype T2 in a human immunodeficiency virus-negative patient. J Clin Microbiol.

[18] Walochnik J, Scheikl U, Haller-Schober EM (2014). Twenty years of *Acanthamoeba* diagnostics in Austria. J Eukaryot Microbiol.

[19] Schroeder JM, Booton GC, Hay J (2001). Use of subgenic 18S ribosomal DNA PCR and sequencing for genus and genotype identification of *Acanthamoeba*e from humans with keratitis and from sewage sludge. J Clin Microbiol.

[20] Booton GC, Kelly DJ, Chu YW (2002). 18S ribosomal DNA typing and tracking of *Acanthamoeba* species isolates from corneal scrap specimens, contact lenses, lense cases and home water supplies of *Acanthamoeba* keratitis patients in Hong Kong. J Clin Microbiol.

[21] Khan NA (2009). *Acanthamoeba*: biology and pathogenesis.

[22] Behera HS, Panda A, Satpathy G, Bandivadekar P, Vanathi M, Agarwal T, et al. Genotyping of *Acanthamoeba* spp. and characterization of the prevalent T4 type along with T10 and unassigned genotypes from amoebic keratitis (AK) patients in India. J Med Microbiol. 2016; doi:10.1099/jmm.0.000234. 10.1099/jmm.0.000234 26887324

[23] Tamura K, Stecher G, Peterson D (2013). MEGA6: molecular evolutionary genetics analysis version 6.0.. Mol Biol Evol.

[24] Liang SY, Ji DR, Hsia KT, Hung CC (2010). Isolation and identification of *Acanthamoeba* species related to amoebic encephalitis and non-pathogenic free-living *Amoeba* species from the rice field. J Appl Microbiol.

[25] Qvarnstrom Y, Nerad TA, Visvesvara GS (2013). Characterization of a new pathogenic *Acanthamoeba* species, A. byersi n. sp., isolated from a human with fatal amoebic encephalitis. J Eukaryot Microbiol.

